# First person to Watch – E. Jennifer Jin, Seungmee Park and Xiaohui Lyu

**DOI:** 10.1242/bio.055780

**Published:** 2020-09-03

## Abstract

First Person to Watch is a series of interviews with the first authors of a selection of papers published in Biology Open, helping early-career researchers promote themselves alongside their papers. E. Jennifer Jin, Seungmee Park and Xiaohui Lyu are researchers in the Neurobiology Section, Division of Biological Sciences, University of California San Diego, La Jolla, CA, USA, and co-first authors on ‘Gap junctions: historical discoveries and new findings in the *C. elegans* nervous system’, published in BiO. E. Jennifer is a postdoc investigating *C. elegans* motor circuit plasticity to understand how transcriptional program and neural activity coordinate developmental neural circuit rewiring. Seungmee is a postdoc investigating regulation of morphological development and maintenance of *C. elegans* neurons, and Xiaohui is a PhD student working on understanding the regulatory mechanism of microtubule dynamics within the *C. elegans* neuron system.


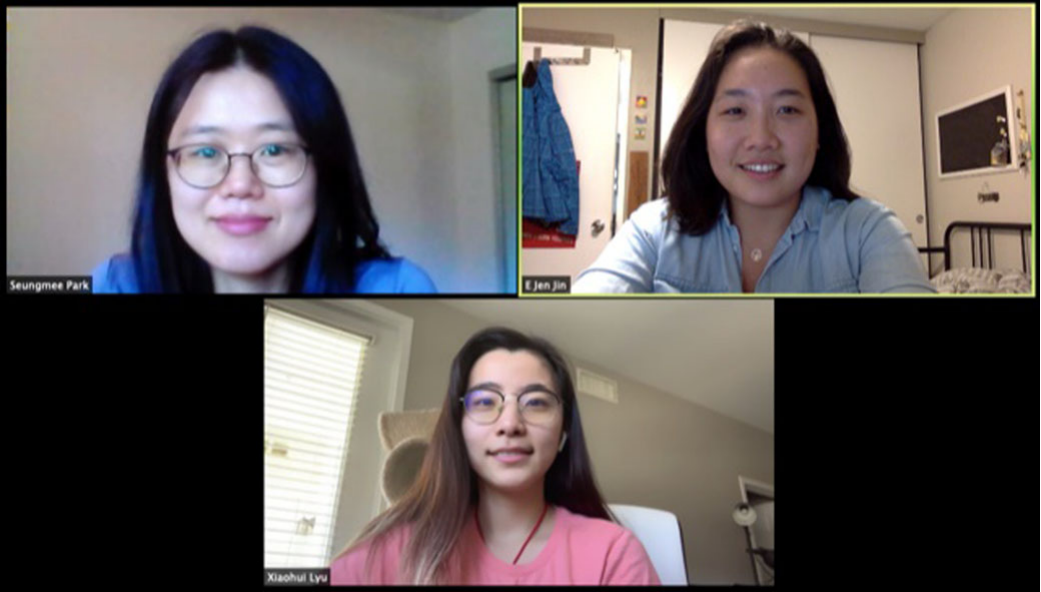


**E. Jennifer Jin, Seungmee Park and Xiaohui Lyu**

**What is your scientific background and the story of how you got to where you are today?**

E.J.J.: My graduate research was to investigate the role(s) of endo-lysosomal function in maintenance of neuronal health in *Drosophila* sensory neurons. For postdoctoral training, I wanted to apply my cell biology research skills to address different sets of neurobiology questions in a simpler genetic model organism. Currently I focus on addressing how neural activity regulates the genetic program of developmental synapse rewiring in *C. elegans* motor circuit. One year into my postdoc training, I still find the transparency of *C. elegans* so beautiful – I could watch worms under DIC all day!

S.P.: I have been always curious about how our cognitive processes are mediated in the brain. It became clear to me that the first step towards satisfying my curiosity is to learn more about fundamental properties of neurons. During my PhD years, I studied two proteins necessary for neuronal communication and my work was geared towards protein structural and functional relationship. For postdoctoral training, I wanted to shift my focus from mechanistic aspects of molecules to regulation of an entire cellular process. Now I am in a ‘hardcore’ genetics lab to identify the genetic basis of regulated network/pathway critical for development and maintenance of neuronal morphology.

X.L.: I graduated from Peking University with a Bachelor of Science degree in Biology. My research experience was mainly on ciliary sensory neurons within *C. elegans*. I have been a PhD student of UCSD Biological Science program since 2018. I really love *C. elegans* as a model organism, and plan to continue to pursue my PhD study using *C. elegans*. I'm currently interested in the regulatory mechanisms of microtubule dynamics in neurons, taking advantage of *in vivo* manipulation of endogenous proteins.

**What is the most important take-home message of your Review?**

In the nervous system, two types of connectivity, chemical synapses and gap junctions (or electrical synapses), play many roles in transmitting signals. While we know a lot about how chemical synapses form and function in a brain, roles and regulations of gap junctions are less well understood. Gap junctions allow direct exchange of small molecules between two cells and gap junction research has focused primarily on how gap junctions regulate neural circuit activity. *C. elegans* also has gap junctions that are functionally equivalent to mammalian gap junctions. The main take-home message we learned from recent studies in *C. elegans* is that gap junctions have roles beyond neural connectivity formation and circuit regulation. For example, gap junctions function in stress responses, neural fate and chemical synapse localization, and gap junction proteins can also function independently of channel opening activity.

**What has surprised you the most while researching this Review?**

It is fascinating that ectopic expression of gap junction genes, which encode proteins called innexins, in two neighboring neurons leads to ectopic gap junction formation and neural circuit rewiring. Even the expression of mammalian gap junction genes, connexins, in two neighboring neurons in *C. elegans*, lead to neural circuit rewiring. This is surprising because in contrast to chemical synapses that have multiple layers of molecular components and steps, gap junction formation seems relatively simple. However, not all neighboring neurons form gap junctions and *C. elegans* neural circuit connectivity is stereotypical, raising the question of how gap junction formation and localization are regulated.

**What do you feel is the most important question that needs to be answered to move the field forward?**

There are many interesting and outstanding questions about gap junctions in the nervous system. Following the previous question, one important outstanding question in the field is how localization, formation and plasticity of gap junctions are regulated. Since brain connectivity is composed of both chemical synapses and gap junctions, understanding the roles and regulation of gap junctions would significantly advance our fundamental knowledge of brain development and function.

**What changes do you think could improve the professional lives of early-career researchers?**

E.J.J.: I think it's important to share stories of success as well as struggles, as we can learn a lot from how one overcame difficult and unprecedented times to move forward with their careers.

S.P.: Writing skills are essential for scientists at any level, but it takes time and effort to improve them. It's important to practice scientific writing as early as possible. Attending workshops and applying for fellowships/grants would help one embark on a journey to acquire excellent writing skills.

X.L.: I think the most important thing is communication with others including principle investigators, senior scientists and also peer researchers. For early-career researchers, experience is something they lack. So efficient communication with different people can help young researchers to learn a lot and go in the right direction.

**What's next for you?**

E.J.J.: Researching for this Review made me want to investigate the roles and regulation of gap junctions in *C. elegans* motor circuit rewiring.

S.P.: I want to complete my on-going projects and publish my work.

X.L.: To finish my doctorate. Since I just started the PhD program 2 years ago, I haven't decided on a future career.

**Share your research experience during the COVID-19 pandemic**

E.J.J.: Working in shifts in the lab has made me much more organized and better at planning my time. I plan to continue this habit into the ‘green phase’.

S.P.: Conducting experiments is not the only part of research. I have learned to write and read in a more meaningful way when I am not in the lab to conduct experiments.

X.L.: Limited time working by the bench actually helps me to organize my time and design experiments in a more efficient way. More time on analyzing and interpreting data also help me to understand my project and know how to keep studying.
